# Oral status of older people in medium to long-stay health and social care setting: a systematic review

**DOI:** 10.1186/s12877-021-02302-x

**Published:** 2021-06-14

**Authors:** Juan Antonio Ruiz-Roca, Dora Martín Fuentes, Francisco J. Gómez García, Yolanda Martínez-Beneyto

**Affiliations:** 1 Department of Gerodontology, Faculty of Medicine-Dentistry, University of Murcia, Avda Marqués de los Velez s/n. Clínica Odontológica Universitaria, Murcia, 30008 Spain; 2 Department of Gerodontology, Research Investigations External, Faculty of Medicine-Dentistry, University of Murcia, Murcia, Spain; 3 Department of Oral Medecine, Faculty of Medicine-Dentistry, University of Murcia, Murcia, Spain; 4 Department of Preventive and Community Dentistry, Faculty of Medicine-Dentistry, University of Murcia, Murcia, Spain

**Keywords:** “Older inpatients”, “older hospitalized patients”, “long term hospitalization”, “long term inpatients”, “Oral health”, “Oral status”

## Abstract

**Background:**

Older patients who spend long periods hospitalized or those who are in a situation of institutionalization represent a risk group in this regard, as many of them suffer a degree of dependence and need help to perform the basic tasks of personal care. It is therefore important to learn more of the oral health status of this group of patients in order to make a proper assessment of the situation and to develop protocols for its management. The purpose of the study was to conduct a systematic review to ascertain the oral health status of older people patients admitted to institutions or hospitalized for a long period of time.

**Methods:**

a systematic review of the literature published in two different databases (PubMed, Embase and Cochrane Library) was carried out, with 12 different combinations of keywords based on the following selection criteria: studies published in the last 5 years, in English and/or Spanish and/or Portuguese, with samples of ≥30 patients, performed in patients older than 65 years, admitted to any type of institution and/or hospital center for at least 7 days and in which the state of hard and/or soft tissues of the oral cavity were evaluated in some way. The selected articles were subjected to a thorough analysis.

**Results:**

The search strategy covered 1.014 articles: 689 from Pubmed and 325 from Cochrane Library. After applying the eligibility criteria, five articles were selected for our review. The level of evidence of the articles was, a sample of 773 patients most of them were women with an average age older than 70 years old.

**Conclusions:**

The oral health of patients aged more than 65 is worse than that of the rest population. Long hospital stays or being institutionalized in a residence makes this group susceptible to a worsening of their oral health status. It is necessary to develop protocols for the oral health care of these patients, accompanied by training programs for the personnel responsible.

## Background

In light of the increase in life expectancy, aging is “on the verge of becoming one of the most significant social transformations of the twenty-first century” [1]. In Spain, people over 65 represent 19.2% of the total population [2], a figure that will reach 25.2% in 2033 [3].

This makes it necessary to reconsider the way in which we attend and treat older patients in society [4], not just those who have sufficient personal autonomy but also those, estimated to represent around 3% of the elderly [5, 6], who live in institutions and need some kind of specific care. Despite this need, there are insufficient studies that describe the situation in which this population group find themselves and which might contribute to improving the attention given to them and therefore increase their quality of life. For example, in Spain there are no studies published in which the physical, medical and psychological conditions of the institutionalized older population are evaluated [5].

The progress and improvements that have been made in dentistry, as well as new patterns of care and prevention, have meant that it is increasingly possible to reach older people with a large number of teeth and in a better state of dentition than ever before [7, 8] although there is still a tendency for the older to be vulnerable to caries and periodontitis [8]. Oral pathologies can significantly affect health and general welfare of the population, and lead to alterations in speech, the poor pronunciation of certain words, or deficient food intake, raising the risk of malnutrition [9] due to problems with chewing or swallowing. Moreover, oral health can have a negative effect on facial aesthetics, lowering self-esteem and harming the psychosocial well-being of the individual [10–12]. Numerous studies have described the relationship between poor oral health and the emergence of systemic diseases, ranging from heart disease or Diabetes Mellitus to respiratory diseases, such as pneumonia [8, 10, 13, 14].

 Diseases such as Parkinson’s or Alzheimer’s, or neuromuscular disorders, are some of the reasons that many are no longer able to carry out oral care tasks, due to a loss of manual dexterity, basically because of a loss of motor and cognitive skill, or because they do not remember how to brush their teeth or are not able to follow the instructions on how to do so themselves [11].

In the case of geriatric patients, the frequent coexistence of several diseases and disorders in the same patient must also be taken into account. Comorbidity in this population makes them especially susceptible to oral pathologies, often as a result of the medication they are taking, which increase the risk of tooth decay through hyposalivation [10]. In addition, some disorders may give rise to physical, cognitive or even motivational limitations that interfere with the development and habit of practicing good oral hygiene [11, 15, 16].

Added to the vulnerability of geriatric patients in this respect, other factors may limit their access to oral attention, such as an inability to assume the costs of treatments reduced physical mobility, the lack of transport or the absence of caregivers or family members who can accompany them. In addition, the work they used to do, their social environment or their own idiosyncrasies may mean the person lacks the ability to recognize the need for an a dental examination or treatment [10].

Despite the high prevalence of oral health problems in this group of patients, little or no importance is given to this problem [10], leading the World Health Organization (WHO) to advise on the need to increase awareness, on a social, cultural and medical level, of oral health as a major component of overall health and quality of life. The organization strongly recommends that countries develop programmes to meet the needs of their older citizens in this respect and to research the problem of oral care in the older people, due to an increase in the overall incidence of non-transmissible diseases [17]. A survey of the oral health of older patients carried out by the WHO revealed that oral health programmes targeting this population group are very rare [17], and that dental intervention tends to be therapeutic rather than (ideally) preventive. That is why hospitalization or long stays in care centers present a good opportunity for providing dental assistance that would otherwise not be offered to the general older population [10].

The removal of bacterial plaque at least twice a day (morning and evening) is essential for maintaining oral health, especially in dependent older people. However, despite the important role that staff in hospitals and other long stay centers such as nursing homes, could play in maintaining and influencing oral health, they do not know what care and oral hygiene protocols should be followed with the older people, except those patients who are at risk of pneumonia associated with mechanical ventilation [11].

Although oral pathologies are among the most common chronic diseases and represent an important public health problem due to their prevalence and the expense of treatment [15], there is a general but erroneous belief that oral hygiene and care are unimportant [11]. When patients, for different reasons, reject oral care, staff simply accept their refusal. However, refusing treatment would not be tolerated in other interventions - for example, measuring the level of glucose in the blood or the blood pressure of a patient. This situation is doubly severe in older patients with dementia who are reluctant to be cared for by third parties, Moreover, care providers may not be in a position to offer proper care, either because the patient refuses or because they are overworked and decide not to assist them. For all of the above, these patients can be considered extremely vulnerable and are at higher risk than the general older population [11].

Bilder et al. [15] describes how poor oral health and limited access to oral care for adults in long-term care centers, as well as the lack of detailed guidelines, are a reflection of insufficient scientific evidence concerning the dental care support techniques that can be offered [11]. This clearly does not help when attempts are made to reverse this situation. However, problems of oral health, ranging from dental caries to chewing problems or pain, constitute the most frequent treatment needs and are among the least successfully resolved health problems in the population group consisting of older people and the disabled [15].

For all these reasons, we think that the lack of information, documentation and prevention concerning the oral health of older patients can have an advese impact on health, i.e., on the state of complete physical, mental and social well-being.

Our main objetive, then, was to conduct a systematic review to ascertain the state of oral health of older patients in an institution for a long period of time, analysing those parameters that could reveal their current oral situation. Secondary objectives were: to see whether any deterioration of oral health detected in these patients is affected by their being in a hospital or residence; to ascertain whether a standard protocol exists concerning the oral health care of these patients; to compare the information obtained with published scientific literature, and, if no relevant information exists, to propose a line of research to establish a prevention-based protocol for oral care in the older population, especially those in long stay facilities.

The literature search strategy followed in making this systematic review was in accordance with the PICOS framework [18]. The focus question was: What is the state of oral health of institutionalized older patients?.

## Methods

### Study design

A systematic review of the literature was managed by two reviewers (JARR and DMF) independently and conducted an exhaustive search of each database.

Following PRISMA (Preferred Reporting Items for Systematic Reviews and Meta-analyzes Statement Check-list) 2009 statements (http: www.prismastatement.org) throughout the selection process and the last manual updated by the Cochrane Collaboration, for the preparation of systematic reviews of the literature of the year 2009″ [19]. Institutional review board approval was not required for this review.

In the first round only titles and abstracts of retrieved articles were analyzed. Then in a second round all considered eligible studies were fully examined and final decisions about inclusions were made. In case of disagreement a third reviewer (YMB) participated in order to reach consensus. Cohen’s kappa coefficient was used to evaluate the disagreement between the researchers.

Following the methodology of evidence-based medicine, the PICO strategy was used, in order to prepare the research question to which we will try to answer in this work; Population (P): older patients, aged 65 and over admitted to hospital or geriatric center for periods of more 7 days; Intervention (I): To analyze the following parameters: Oral health indexes such as DMFT (Decayed, Missing, Filled Index) and treatment needs index and oral hygiene protocols Comparison (C): Oral health status among patients who are institutionalized versus non- older subjects; Outcome (O): Poorer results in patients institutionalized in the periodontal index score.

### Search strategy and databases

An intensive search was performed in three of the main scientific databases such as the Cochrane Library, Medline via Pubmed and Embase. Only articles published in English, Spanish or Portuguese within the 5 year period 1 January 2014 to 1 January 2019 were consulted. The search strategy used terms from the controlled vocabulary MeSH (Medical Subject Headings) and the Boolean operators “AND”, “OR” and “NOT”, as well as terms related to the study population (*elderly inpatients, elderly hospitalized patients, long term hospitalization, long term inpatients, oral health oral status and oral pathology)*.

### Selection criteria

The following inclusion and exclusion criteria were followed in this systematic review (Table 1). The sample was ≥50 individuals, because that amount of sample is statistically representative, usually. In Spain, an older patient is considered to be ≥65 years old.

**Table 1:** Inclusion and exclusion criteria

INCLUSION CRITERIA	EXCLUSION CRITERIA
Studies published in the last 5 years	Not published un english, spanish or portuguese language
Case-control, cross-sectional, longitudinal and cohort studies	Systematic Review, Meta-Analysis, Case Report, Letter, Editorial, Congress
Humans	Children, teenagers and/or non-older adults
Older patients ≥65 years old	Studies in which the essential data are missing in order to obtain a profile of homogeneous works
Sample ≥ 50 individuals	Studies whose access to the complete text was under private subscription
Entered in some type of institution or hospital center for a period ≥7 days	
In which the situation of hard and/or soft tissues the oral cavity was evaluated in some way	

### Assessment of Bias in studies

From each of the articles, information was extracted, such as sample size, study design, any intervention and the measures of the results, how the results were measured/analysed/presented?. Articles are classified by reference to their level of scientific evidence according to the criteria described by the Scottish Intercollegiate Guidelines Network (SIGN), which provides checklists to assess the quality of: systematic reviews & meta-analyses, randomized clinical trials, cohort studies, case-control studies, diagnostic studies, and economic studies. Each checklist is accompanied by notes to aid completion, and written responses to the individual questions are used, with users then assigning studies an overall rating according to specified criteria. The full set of checklists and detailed notes on their use are available from SIGN [19].

## Results

**Fig. 1:**
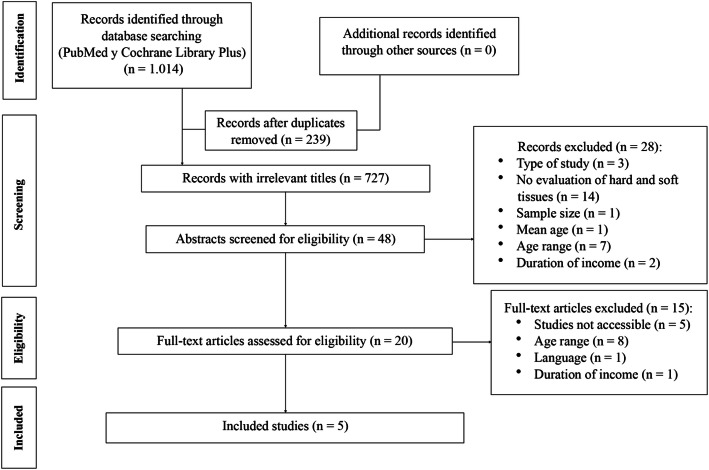
Flow diagram of the search processes and results

The search culminated in five studies that fulfilled both the inclusion and exclusion criteria and which were conducted from 1 January 2014 to 1 January 2019 (Fig. 1).

### Quality assessment

According to these criteria, the articles selected for our systematic review had the following levels of evidence and degrees of recommendation and the Risk of bias for non-randomized studies assessed with ROBINS-I Cochrane tool. (Tables 2 and 3).

**Table 2:** Risk of bias for non-randomized studies assessed with ROBINS-I Cochrane tool

Risk of Bias domains included in ROBINS-I Cochrane tool.	Category of BIAS	Poisson et al.	Gerritsen et al.	Chen et al.	Nakayama et al.	Murray et al.
**Pre-intervention domains**						
1.Bias due to confounding.	Confounding	3	2	1	2	3
2. Bias in selection of participants into the study.	Selection Bias	3	1	1	2	2
**At-intervention domain**						
3. Bias in classification of interventions.	Information bias	1	1	1	1	2
**Post-intervention domains**						
4. Risk of bias due to deviations from the intended interventions (effect of assignment to intervention)	Confounding	2	2	1	1	1
5. Bias due to missing data	Selection Bias	2	2	1	1	1
6. Bias in measurement of the outcome	Information bias	1	1	1	1	1
7. Bias in selection of the reported result	Reporting bias	2	1	1	1	1
**Overall Risk of BIAS for the results**		**3**	**2**	**1**	**2**	**3**

**Table 3:** Quality assessment and general characteristics of included studies

AUTHOR	STUDY DESIGN	LEVEL OF EVIDENCE	DEGREES OF RECOMMENDATION	SAMPLE	MEAN AGE	GENDER	TYPE OF CENTER	REASON FOR ADMISSION	DURATION OF INCOME	SYSTEMIC PATHOLOGY
Poisson et al. [9]	Cross sectional	3	D	159	85.28	51 M 189F (1:2)	Hospital	–	17.2 days	74.2% cognitive disease
Gerritsen et al. [20]	Cohort	3	D	355	84.1 ± 6.9	110 M 245F (1:2.3)	3 Nursing homes	Somatic (47%), psychogeriatric (53%)	2.3 ± 2.6 years	Unspecified
Murray et al. [21]	Cohort	3	D	89	74	57 M 32F (1.7:1)	3 Rehabilitation homes	CVA (cerebrovascular accident)	≥ 7 days	Co-morbilities of CVA (aphasia, apraxia, …)
Makayama et al. [22]	Cross sectional	3	D	50	70.7	31 M 19F (1.6:1)	Hospital	ALE	≥ 7 days	ALE
Chen et al. [23]	Case control	3	D	120	80.28	52 M 68F (1:1.3)	Geriatric medical center	Pneumonia, sepsis, urinary tract infection, idiopathic fever	7–10 days	Diabetes (58.3%), arterial hypertension (77.5%), dependence (45% total; 35% serious; 20% light)

### Basic results

Of the five studies selected for this systematic review (Fig. 1), two were carried out in Europe [9, 20], two in Asia [21, 22], and one in Australia [23].

All the works were based on with a sample size that could offer extrapolated data (≥ 50 older patients): Poisson et al. [9] 159 patients, Gerritsen et al. [20] 355, Chen et al. [21] 120, and that smallest, Murray et al. [23] and Nakayama et al. [22] with 89 and 50 patients, respectively, making a total of 773 patients.

Although only two studies [20, 21] specified the age range of the patients, the average age of all participants in the studies was over 70 years.

In three studies [9, 20, 21] the proportion of women in the sample was higher than that of men. As regards the total number of participants in the papers included in the review, the proportion of women who participated in the studies was higher (1:1.6 male to female ratio), which can be explained by the greater life expectancy of women.

Gerritsen and co-workers [20] took as their sample a group of older subjects from three retirement homes, while Chen et al. [21] studied subjects from a geriatric medical center and Murray et al. [23] patients from three rehabilitation centers for patients who had suffered a cerebrovascular accident (CVA). Poisson et al. and Nakayama et al. [9, 22] developed their studies in hospitals, and Poisson et al^.^ [9] worked in the geriatrics area of a hospital. Nakayama et al. [22] focused on patients suffering ALS (Amyotrophic Lateral Sclerosis) with nasogastric and artificial respiration. None of the selected studies specified whether they were in public or private centers.

### Causes of admission of patients

Except for Murray et al. [23] and Nakayama et al. [22], who worked with very specific types of patient (patients in rehabilitation after CVA and patients with ALS, respectively), none of the studies specified the reason for admission to the centres, although Gerritsen et al. [20] and Chen et al. [21] gave a general outline. In particular, Gerritsen et al. [20] specified that 47% were in the residence for somatic reasons and 53% for psychogeriatric reasons, while Chen et al. [21] pointed out that the main diagnoses of their sample at admission were pneumonia, sepsis, idiopathic fever and infection of the urinary tract.

Two of the studies [22, 23] did not specify the length of the stay in the institution, but, from the information provided in the articles, we understand that all the studied patients had been in institution for at least 7 days [21, 23], while the longest times were those mentioned by Gerritsen et al. [20] (more than 2 years). Therefore, the subjects who had been the longest time in care were those mentioned in the only study carried out in retirement homes.

Three of the five studies [9, 21, 23] specify at least part of the systemic pathology that participants were suffering. The remaining two [20, 22] did not mention whether the patients described in their studies suffered any other pathologies beyond those specified as the time of admission: somatic or psychogeriatric reasons in the case of Gerritsen et al. [20], and ALS in the case of Nakayama et al. [22]. In the study of Poisson et al. [9], 74.2% of the patients had some sort of cognitive problem. Murray et al. [23] mentioned only comorbidities derived from the CVA suffered by their patients (aphasia, apraxia, dependency, among others) and Chen et al. [21] describes the degree of dependence of their patients (total 45%; severe 35% and slight 20%), along with the more common pathologies such as Diabetes Mellitus (58.3% of patients) and high blood pressure (77.5%). However, the most striking thing in all the studies was the number of patients who had some sort of cognitive problem or degree of dependence that made them vulnerable if they did not receive good oral care (Table 3).

None of the studies evaluated the medication that the participants were taking despite the fact that medication could be associated with the state of their oral health. Nakayama et al. [22], who measured the salivation index of their participants, only mentioned that none of the patients in the study were following any treatment that would have affected their salivary flow (radiotherapy or botulinum toxin treatment).

### Oral health and hygiene

Regarding the oral health of the participants in the studies, we conclude that the authors used different methods of assessment, and only Poisson et al. [9] and Nakayama et al. [22] used the DMFT index (Decayed, Missing, Filled Teeth). However, the vast majority of patients in all the studies had poor oral health and, we understand that they were also in great need of treatment, although only Gerritsen et al. [20] specified so.

As regards oral care measures, only one study [9] did not mention that subjects follow any kind of oral hygiene protocol. Gerritsen et al. [20] mentioned that patients in the caring homes had access to 16 h of dental care a week and 8 h of oral hygiene. This is probably why new patients had greater need of treatment than long-standing residents, although this relationship was only clear in the group of edentulous patients. This fact is possible due to they had no teeth, and this made it easier to offer care and because their mental condition meant they have received special attention. Nakayama et al. [22] described the protocol followed by nurses twice a day, in which they paid attention to both the hard and soft tissues. However, it must be borne in mind that the patients who participated in the study by these authors suffered from ALS, suggesting that they followed a special protocol (even though, in our opinion, such care should be considered normal). Chen et al. [21] suggested that the oral hygiene of patients is the responsibility of the nursing staff, but did not specify any guidelines or the frequency concerning the same. However, the authors do mention the improvements shown following the intervention (brushing and rinsing twice a day) with regard to halitosis, plaque and the state of mucous membranes. No significant differences were observed between the three types of rinses used for the different groups (Chlorhexidine, saline solution and boiled water) during the examinations carried out on the 7th day of the intervention, except for cases of halitosis, for which the best result was seen in the.

In the case of Murray et al. [23] it seems that patients only had their teeth brushed in the morning but that, due to the hygiene guidelines provided during the study (brushing with toothpaste after breakfast and dinner, and rinsing with water after the main meal, with the assistance of the staff when necessary), the oral situation of most of the patients with dysphagia improved; patients without dysphagia also improved, but not significantly so. In addition, the authors established a relationship between patient autonomy and their oral status. Improvement in the oral health of patients were recorded in the only two studies that provided oral hygiene guidelines during the studies and reassessed the oral situation of patients later [23]. It should be noted that only the studies of Poisson et al. and Gerritsen et al. [9, 20] were supervised by dentists (Table 4).

**Table 4:** General characteristics of included studies and statistical significance.(*significant at p < 0.05)

AUTHOR	MEDICATION	ORAL HEALTH ASSESSMENT	GUIDELINES OF ORAL HYGIENE (1)	GUIDELINES OF ORAL HYGIENE (2)	***p-value***
Poisson et al. [9]	NO	DMFT (20.2) Need for treatment (89.3%)	NO	NO	*p* = 0.004* (autonomy for oral care vs not autonomy)
Gerritsen et al. [20]	NO	Need for treatment (70%)	Oral hygiene 8 h/week	NO	*p* = 0.053
Murray et al. [21]	NO	OHAT (dysphagia 4 (0–10) // not dysphagia 2 (0–8)	1 brushing in the morning	Brushing toothpaste (after breakfast and dinner), rinses with water (after lunch)	dysphagia *p* = 0.024* not dysphagia *p* = 0.282
Makayama et al. [22]	Decrease salival flow (botix, …)	DMFT (13)	Twice in a day	NO	*p* > 0.05
Chen et al. [23]	NO	Own index reviewed by 2 dentist and one nurse to measure halitosis, bacterial plaque and mucosal status	Assisted by nurses (does not specify	10–15 min. After lunch and after dinner with toothbrush and 3 different mouthwashes: clorhexidine, saline solution and boiled water	*p* = 0.002*

In general, the studies included in our systematic review [9, 20, 3] found that the attention that should be given to the hygiene and oral care of patients is simply not given, and that staff, by implementing measures that are considered basic for maintaining good oral health, could improve the oral health of many people in this population.

## Discussion

### Relationship between oral and general health

There is no doubt that a good oral health status is crucial for maintaining good general health [8, 10, 13]. In the older this relationship is much clearer, since many tend to suffer from conditions that make them susceptible to poorer oral health [10, 11, 15].

In a study carried out in 2001, Shimazaki et al. [24] showed that older edentulous subjects not using dentures were significantly (*p* < 0.05) associated with hight risk of physical disability and mortality, independent of age and other variables (OR = 1.8, 95% CI). The decline in occlusal function resulting from tooth loss causes problems with chewing, swallowing, an food selection, and the nutritional status of edentulous people deteriorates. Therefore, Shimazaki et al. [24] concluded that those older inpatients with 20 or more teeth, leads to think that the conservation of teeth as the years pass exerts a protective role in the general state of health. These same authors studied the influence of oral health on febrile states in older inpatients during long hospital stays, and found that poor dental and oral health was linked to episodes of fever in both dentate and edentulous patients. In addition, many authors have described the relationship between poor oral health and the development of pneumonia as a result of aspiration and respiratory infections in patients with assisted ventilation [16]. This suggests that, while dental conservation work can favour the maintenance of a good general state of health in old age, the same does not apply if little attention has been paid to maintaining oral health previously.

### Impact of hospitalization

Hospitalization changes the routines of people, and may cause stress or anxiety because of the pain and discomfort that they may experience during an illness [25]. For this reason, being hospitalized is an added risk when it comes to good oral health [16], as it usually results in a decline in self-esteem, leading patients to neglect personal care and hygiene at that same time that they feel worried about their disease [16, 25]. This circumstance particularly affects patients with physical or cognitive limitations [12, 25–27], who are the most vulnerable in terms of developing problems or deterioration in terms of oral health, especially during a long hospital stay or a situation of institutionalization.

### Lack of data on longer term institutional care settings

Studies that have attempted to look for a relation between hospitalization and oral health were developed in Intensive Care Units (ICU), and so provide insufficient evidence since the vast majority of hospitalized patients attend other departments [25]. In addition, Sousa et al. [25] and Gibney et al. [16] found that short hospital stays in units that did not involve intensive care had a negative effect on the oral health of patients, corroborating the evidence of studies conducted in these units, and underlining the importance of studying the situation in other hospital services.

During the article selection process we were faced with the problem of the paucity of studies on elderly institutionalized or long-stay hospital patients and their oral health, although many authors [10, 16, 28] studied emergency and short-stay patients, who were found to have previous oral health as well as systemic problems. Other studies focused on dysphagia which elderly patients frequently suffer, its risk factors and relationship with malnutrition, but without analyzing the state of their oral health [29–32], despite its importance in this disorder. In our review [9, 20–22], we observed that the vast majority of participants had poor oral health. For example, Gerritsen et al. [20] established the need for treatment in 70% of the patients in their sample, even though dental care was provided by their institution, which makes the lack of studies assessing the oral health of the older in this situation or during long hospital stays even more incomprehensible.

### The need for standardize protocols

As we have seen in two of the studies included in our review [21, 23], compliance with the protocols that involve the basic oral hygiene measures recommended for any patient these days leads to unquestionable improvements in the oral health of patients. This has also been found in other studies carried out in chronic care facilities and in areas of geriatric rehabilitation where oral hygiene measure were under the supervision of dental professionals and/or nursing staff following a standardized protocol [28].

Health care personnel recognise the importance of hygiene and oral care [33]. However, the lack of such care in long stay institutions and hospitals [34] is frequently attributed to a lack of training and time and the little cooperation of geriatric patients themselves [33]. Many studies have pointed to the difficulty posed by applying protocols of oral hygiene in institutions such as old people’s homes [20] due to the little training received by care workers concerning protocols of oral hygiene, the oral needs of older patients, and the risks and negative consequences of poor oral health [15, 16], as well as on the availability of and access to material to carry out related tasks [11, 35]. In addition, it has been described how a theoretical training programme is not sufficient to improve the oral care of these patients. In this context, Gammack et al. [36] found that when hygienists, auxiliary staff and nurses were given oral hygiene training on a theoretical basis using audiovisual aids and dummies rather than “real” patients, the oral health of dependent patients did not improve, perhaps because, among other reasons, staff had not received adequate training or information on the correct way to deal with the reactions of patients opposed to receiving much care [28]. However, some studies suggest that the attitude of the staff themselves towards providing oral care makes the difference between a patient accepting, asking for or neglecting oral care [11]. It is clear that oral health is not a priority in situations of lengthy hospitalization or institutionalization [11, 37]. As we have seen in the results of the review, only one study [22] presented a detailed oral hygiene protocol to be applied twice a day, although, being a protocol used in patients with ALS, we understand that this is a special feature because of the medical condition in question.

### The situation in Spain

In Spain, according to National Oral Health Surveys, carried out in 2015, the 20% of the population over 65 years old, worry less about their oral health, and visit the dentist less frequently [38]. Perhaps, for this reason, in the group of 65–74 years, the SIC (Significant Caries Index) of Bratthall, represents the highest value, a 25.27 ± 2.80, compared to the adult population (34–44 years) or adolescent population of 15 years old, whose values are 14.29 ± 3.86 and 3.73 ± 2.11, respectively [39].

Despite this situation, and the greater risk of developing oral pathologies as mentioned above, the number of complications and problems that can occur in this population group due to deficient oral health, there are no specific programmes dedicated to the prevention or promotion of oral health in the older population in Spain. In the published scientific literature, we only found one study dedicated to the development of a geriatric dental care programme (PADGE, in its Spanish acronym) [40] developed in the Public University of Navarra (Spain).

However, as in many cases the proposed programme remained just a proposal, and to this day remains to be implemented even at a regional level, and this in a Autonomous Community regarded as being a leader in preventive oral health programs. It seems that neither governmental nor local authorities consider worthwhile the logistic and economic effort that such a programme would involve. Population aging and the poor dental state of many people over 65 years of age, accompanied by a lack of specific studies and the quality of those that exist (the studies included in our systematic review had a level of evidence of 3 and grade of recommendation D, according to the SIGN criteria) on long term stays in hospitals and other institutions, together with the lack of protocols for promoting good oral hygiene and health care in nursing homes and hospitals, underline the importance of this line of study in the future. For this reason, and due to the lack of time available to develop the present overview, we intend to expand the study by developing a universal protocol for dental care in institutionalized patients.

## Conclusions

The oral health of older patients aged over 65 years, whether hospitalized for long periods of time or living in institutions, is deficient, and a homogeneity in methodology of studies are needed. Furthermore, theoretical and practical continuous training courses would be necessary with the aim of training caregivers in oral health techniques.

## Data Availability

Data and materials are available ordering to the corresponding author.

## Abbreviations

WHO: World Health Organization
DMFT: Decayed, Missing, filled teeth
JARR: Juan Antonio Ruiz Roca
DMT: Dora Martin Fuentes
YMB: Yolanda Martínez Beneyto
FJGG: Francisco Jose Gomez Garcia
SIGN: Scottish Intercollegiate Guidelines Network
RCT’S: Ramdomized Controlled Trials
CVA: Cerebral vascular accident
ALS: Amyotrophic Lateral Sclerosis
ICU: Intensive Care Unit
SIC: Significant Caries Index
PADGE: Geriatric Dental Care Programme
OR: Odds Ratio
CI: Confidence Interval
DMFT: Decayed, Missing, Filled Index
